# Filariae-Retrovirus Co-infection in Mice is Associated with Suppressed Virus-Specific IgG Immune Response and Higher Viral Loads

**DOI:** 10.1371/journal.pntd.0005170

**Published:** 2016-12-06

**Authors:** Kirsten Katrin Dietze, Ulf Dittmer, Daniel Karim Koudaimi, Simone Schimmer, Martina Reitz, Minka Breloer, Wiebke Hartmann

**Affiliations:** 1 Institute of Virology, University Hospital Essen, University of Duisburg-Essen, Essen, Germany; 2 Bernhard Nocht Institute for Tropical Medicine, Hamburg, Germany; George Washington University, UNITED STATES

## Abstract

Worldwide more than 2 billion people are infected with helminths, predominantly in developing countries. Co-infections with viruses such as human immunodeficiency virus (HIV) are common due to the geographical overlap of these pathogens. Helminth and viral infections induce antagonistic cytokine responses in their hosts. Helminths shift the immune system to a type 2-dominated immune response, while viral infections skew the cytokine response towards a type 1 immune response. Moreover, chronic helminth infections are often associated with a generalized suppression of the immune system leading to prolonged parasite survival, and also to a reduced defence against unrelated pathogens. To test whether helminths affect the outcome of a viral infection we set up a filarial/retrovirus co-infection model in C57BL/6 mice. Although Friend virus (FV) infection altered the *L*. *sigmodontis*-specific immunoglobulin response towards a type I associated IgG2 isotype in co-infected mice, control of *L*. *sigmodontis* infection was not affected by a FV-superinfection. However, reciprocal control of FV infection was clearly impaired by concurrent *L*. *sigmodontis* infection. Spleen weight as an indicator of pathology and viral loads in spleen, lymph nodes (LN) and bone marrow (BM) were increased in *L*. *sigmodontis*/FV-co-infected mice compared to only FV-infected mice. Numbers of FV-specific CD8^+^ T cells as well as cytokine production by CD4^+^ and CD8^+^ cells were alike in co-infected and FV-infected mice. Increased viral loads in co-infected mice were associated with reduced titres of neutralising FV-specific IgG2b and IgG2c antibodies. In summary our findings suggest that helminth infection interfered with the control of retroviral infection by dampening the virus-specific neutralising antibody response.

## Author Summary

The coincidental infection of a host with two different pathogens is widespread in low-income countries. Regions where helminth infections are endemic strongly overlap with areas where the incidence of viral infections such as HIV is high. HIV is a major public health issue causing more than 1 million deaths per year. To analyse the impact of a pre-existing helminth infection on a viral infection we established a helminth/retrovirus co-infection mouse model. Mice that were first infected with *Litomosoides sigmodontis* and subsequently with a murine retrovirus showed a more severe course of virus infection, i.e. exaggerated splenomegaly and higher viral loads. Since different lymphocytes such as B and T cells contribute to viral control we analysed the cellular and humoral immune response. While T cell responses were similar in co-infected and virus-infected mice, we observed reduced titres of virus-specific antibodies in co-infected mice. Our results suggest that helminth infection interfered with viral control by dampening the virus-specific antibody response. The viral infection itself altered the humoral immune response against *L*. *sigmodontis* without changing the worm burden. In summary, our data highlight the importance of deworming programs or vaccines against helminths in developing countries where the incidence of helminth/HIV co-infections is high.

## Introduction

One third of the world population is infected with helminths [1]. Helminth endemic areas overlap with high-risk areas for viral infections in the developing countries [1–3]. Interestingly, antagonistic immune responses control helminth and viral infections. Viral infections skew the immunity towards a proinflammatory type 1 immune response, while helminths strongly polarize towards a type 2 cytokine response [4]. In addition helminths are known for their capacity to dampen the immune response directed against them. Helminth-induced immunosuppression is mediated by the induction of regulatory cell types such as regulatory T (Treg) and B cells, negative regulatory receptors such as cytotoxic T lymphocyte associated protein-4 [5, 6], B and T lymphocyte attenuator [7] and programmed death-1 [8], and anti-inflammatory cytokines such as interleukin-10 and transforming growth factor-β [9]. This helminth-mediated downregulation of immunity affects immune responses to unrelated ‘third party’ antigens and thus can be detrimental for the host in terms of vaccinations or co-infections [9, 10]. Indeed, several human studies suggest that helminth co-infections worsen the outcome of a virus infection [10]. For instance, individuals infected with soil-transmitted helminths, filarial nematodes or water-borne schistosomes, were more susceptible to infection by the human immunodeficiency virus (HIV) [11, 12], hepatitis C virus [13] and human papillomavirus [14] and/or suffered from increased pathology [13, 15]. Drug-induced deworming decreased HIV loads [11, 16–18] in some studies, while anthelminthic treatments had no beneficial effect on HIV infection in other studies [19, 20]. The mechanism underlying helminth-mediated suppression of virus control was not identified in these studies. To analyse helminth-virus co-infections we set up a co-infection model by first infecting C57BL/6 mice with *Litomosoides sigmodontis*, followed by infection with FV. Infections of mice with FV are used to study immunity against retrovirus infections [21, 22]. FV is a murine retroviral complex that consists of the apathogenic replication-competent Friend murine Leukaemia Virus (F-MuLV) and the pathogenic but replication-defective Spleen Focus Forming Virus (SFFV). Initial replication of FV takes place in infected erythroid progenitor cells followed by the infection of B cells, T cells, and monocytes/granulocytes [23]. Susceptible BALB/c mice suffer from splenomegaly and succumb to infection due to erythroleukaemia within a few weeks. Resistant C57BL/6 mice generate an immune response to protect them sufficiently from lethal leukaemia, but are not able to eradicate the virus completely and a life long persistence of the virus develops [24]. Similar to infections with HIV, this retroviral complex is controlled by B cell responses, virus-specific cytotoxic T lymphocytes and CD4^+^ T cells [21, 25]. CD4^+^ T cells either act as effector cells or most likely as T helper cells for CD8^+^ T cells and the production of high-affinity antibodies by B cells [26]. Neutralising antibodies are crucial for the control of FV during acute infection and for vaccine-induced protection against FV [27–30].

Infections of mice with *L*. *sigmodontis* are commonly used as a model for human filariasis displaying all features of type 2 immune responses [21] and immunomodulation observed in infected humans [31, 32]. Infective third stage larvae are transmitted by blood-sucking mites, *Ornithonyssus bacoti*, and migrate via the lymphatic system to the pleural cavity. There, they moult to fourth-stage larvae (L4) within 10 days and to immature adults within 28 days. In susceptible BALB/c mice mature adults mate and females release their offspring, named microfilariae, into the circulation by day 60 post infection (p.i.). C57BL/6 mice are semi-susceptible to infection with *L*. *sigmodontis*. This mouse strain is also efficiently infected, but the parasites do not reproduce and are eradicated within 60 days [33]. We previously reported that *L*. *sigmodontis* infection suppressed B and T cell responses to unrelated antigens in both, BALB/c [34, 35] and C57BL/6 mice [36, 37]. Strikingly, the suppression of bystander immune responses did not require an acute *L*. *sigmodontis* infection. Suppressed B cell responses were still observed 16 weeks after the release of microfilariae into the peripheral circulation had stopped and thus most likely after the eradication of *L*. *sigmodontis* female adults [34]. Thus, C57BL/6 and BALB/c mice offer a suitable model to study helminth-mediated immune suppression. The current study had to be performed in semi-susceptible C57BL/6 mice because FV does not induce measurable immune responses in BALB/c mice and rapidly kills infected animals.

Here, we show a more severe splenomegaly and enhanced viral loads in *L*. *sigmodontis/*FV-co-infected mice compared to FV infection alone. *L*. *sigmodontis* infection neither changed the numbers of FV-specific CD8^+^ T cells nor the cytokine response by CD4^+^ and CD8^+^ T cells. Likewise, the phenotype of CD4^+^ T cells and the number of Foxp3^+^ Treg were similar in co-infected and FV-infected mice. However, *L*. *sigmodontis* infection resulted in significantly reduced FV-specific IgG2b/c titres and FV-neutralising Ig responses. On the other hand, FV infection altered the *L*. *sigmodontis*-specific humoral immune response without having an effect on the worm burden. Thus, our results suggest that *L*. *sigmodontis*-induced interference with the FV-specific humoral immune response contributed to the impaired virus control in helminth/retrovirus co-infected mice.

## Materials and Methods

### Ethic statements

Animal experimentation was conducted at the animal facility of the Bernhard Nocht Institute for Tropical Medicine in agreement with the German animal protection law. The experimental protocols have been reviewed and approved by the responsible federal health Authorities of the State of Hamburg, Germany, the "Behörde für Gesundheit und Verbraucherschutz" permission number 44/14.

### Mice, pathogens and experimental infections

Female C57BL/6 mice were purchased from Harlan and kept in individually ventilated cages. The *L*. *sigmodontis* life cycle was maintained in infected cotton rats as described before [37]. Eight to 10 week old C57BL/6 mice were naturally infected by exposure to infected mites (*Ornithonyssus bacoti*) that had been infected 14 days earlier. Mice from different groups (*L*. *sigmodontis*-infected and co-infected) were placed anesthetised on the same sawdust with infected mites. The FV stock used in these experiments was a FV complex containing B-tropic F-MuLV and polycythemia-inducing SFFV [38]. The stock was prepared as described before [39]. Mice were infected intravenously with 1.5 x 10^4^ SFFU of FV spleen homogenate. Mice were sacrificed by deep CO_2_ narcosis at day 20 post FV infection. Worms were counted after flushing the thoracic cavity with 10 ml cold PBS. Spleen, BM and LN (popliteal, inguinal, cervical, and axillary) cells were isolated. The infectious centre assays were performed as described previously [40]. In the vice versa experiments mice were first infected for 24 days with FV and then superinfected with *L*. *sigmodontis* for a further period of 25 days.

### Flow cytometry

For IFN-γ and IL-4 production, cells were restimulated for 5 h with 2 μg/mL anti-CD28, 10 μg/mL plate-bound anti-CD3 and Brefeldin A. Cells were stained with Live/Dead Fixable Blue Dead Cell Stain Kit (Life Technologies) or Fixable Viability Dye eFluor® 450 (Affymetrix eBioscience) according to the manufacturers’ instructions. For surface staining, cells were stained with anti-mouse CD4-Alexa Fluor (AF) 680 (clone: RM4-5), anti-mouse CD8-AF488, -Allophycocyanin, -PE-Cy7 (clone: 53–6.7), anti-mouse CD43 PE-Cy5 or PerCP (clone: 1B11) for 30 min on ice. For detection of D^b^-GagL-specific CD8^+^ T cells, cells were stained with PE-labelled MHC class I H2-D^b^ tetramers (Tet) specific for FV GagL peptide. For intracellular expression, cells were stained with anti-mouse IL-4 PE/Cy7 (clone: 11B11) and INF-γ Texas Red or AF488 (clone XMG1.2), or anti-mouse/anti-rat Foxp3 (clone: FJK-16s)-staining Set (Affymetrix eBioscience) according to the manufacturer's instructions. Gating strategy is shown in supplementary Fig 1 (S1 Fig). Antibodies were purchased from BioLegend or Affymetrix eBioscience. Samples were measured on a LSRII Flow Cytometer (Becton Dickinson) and analysed using FlowJo software (TreeStar).

**Fig 1:**
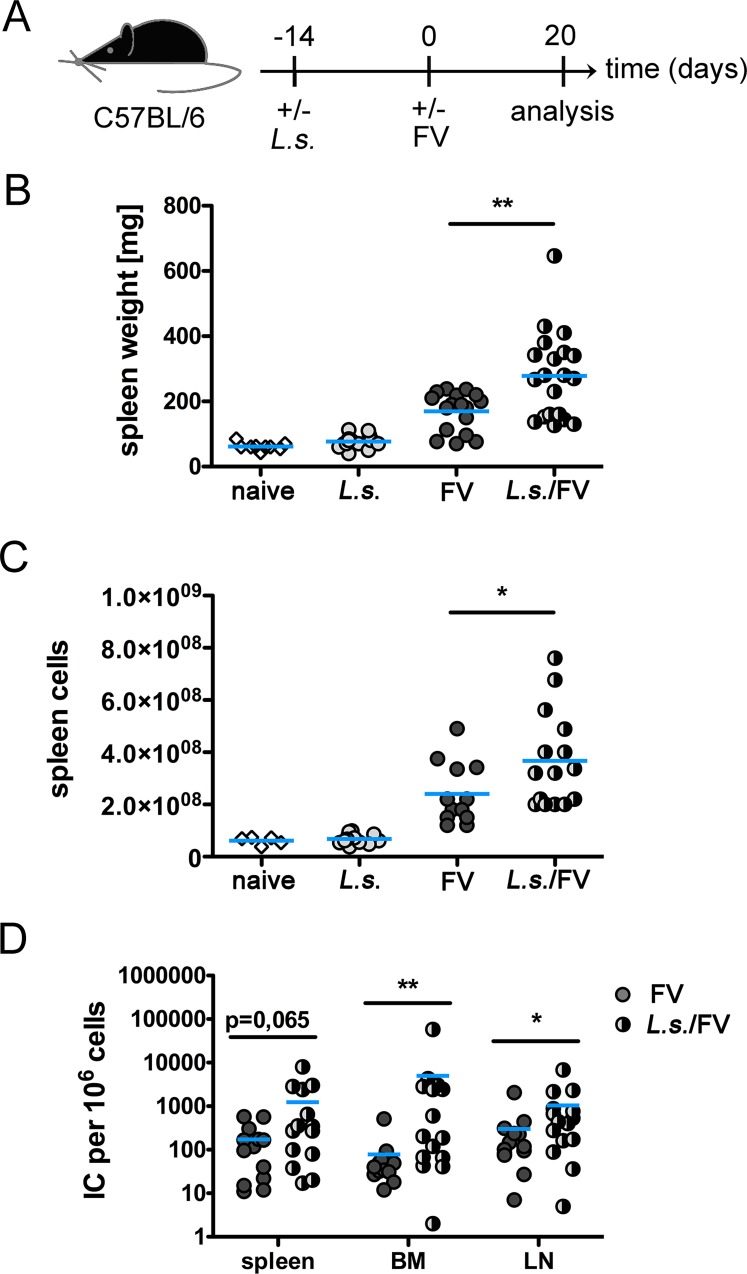
Increased splenomegaly in co-infected mice is associated with higher viral loads. A) Experimental Setup: C57BL/6 mice were infected for 14 days with *L*. *sigmodontis* (*L*.*s*.) and superinfected with FV for additional 20 days. Control groups were either left uninfected or infected with *L*. *sigmodontis* or FV only. Spleen weights (B) and number of spleen cells (C) at day 20 p.i. (D) Viral loads were determined in spleen, BM, and LN cells. Each data point represents an individual mouse. Data are combined from 2–4 experiments (n = 2–5 mice per group and experiment). The line shows the mean and statistical significances are indicated between the groups (*p ≤ 0.05, **p ≤ 0.01).

### Quantification of the humoral immune response

ELISA plates were coated overnight with 5 μg/mL F-MuLV antigen or 4 μg/mL *L. sigmodontis* Antigen (*Ls*Ag) in PBS. FV-specific IgG2 was measured and calculated as described before [37]. FV-specific isotypes were defined as the highest serum dilution in a serial dilution (1:100 to 1:6.400 for IgG2b and 1:1000 to 1:64.000 for IgG2c) resulting in an OD_450nm_ above the doubled background. The background OD_450nm_ of the diluent (0.1% BSA in PBS) was always below 0.1. For detection of *Ls*Ag-specific Ig, sera were either titrated or diluted in a fixed serum concentration as described before [34]. For the analysis of FV-neutralising antibodies, heat-inactivated sera were mixed with an equal volume of 0.2 M β-mercaptoethanol (ME), incubated for 30 minutes at 37°C, and then serially diluted (1:10 to 1:640) with PBS 0.01 M β-ME. Sera were mixed with purified F-MuLV and guinea pig complement (Sigma-Aldrich). After 1 h incubation at 37°C samples were added to *M*. *dunni* cells which were plated in 24-well plates the day before at a density of 7.5 x 10^3^ cells per well. Infectious centre assays were performed as described previously [40]. Foci were counted and dilutions that resulted in a reduction of foci number by 75% or more were considered neutralising. The titre of sera that did not inhibit FV infection in a 1:10 dilution was defined as 1.

### Preparation of *Ls*Ag

*Ls*Ag was prepared by homogenization of agile male and female worms isolated from infected BALB/c mice, followed by centrifugation at 10.000 x g for 30 min at 4°C. The supernatant was passed through a 0.22-mm filter and then stored at -80°C until use.

### Serum transfer

Blood was obtained from naïve, *L*. *sigmodontis*-infected, FV-infected and co-infected mice at day 18 by cardiac puncture and allowed to coagulate for at least 1 h at RT. Serum was collected after centrifugation at 10.000 x g for 10 min and stored at -20°C. Recipient C57BL/6 mice were infected with FV as described. At day 3 p.i. 250–300 μl serum (derived from naïve, *L*. *sigmodontis*-infected, FV-infected, or co-infected mice) were intraperitoneally injected. Mice were sacrificed at day 7 post FV infection and viral loads were determined in the BM.

### Statistical analysis

Samples were tested for Gaussian distribution and students *t* test (unpaired) or Mann-Whitney test were performed to compare 2 groups. 1-way Anova with Bonferroni post-test or Kruskal-Wallis with Dunn`s multiple comparison test were performed to compare more than 2 groups. Prism software was used for statistical analysis (GraphPad Software). P-values ≤ 0.05 were considered statistically significant.

## Results and Discussion

To analyse helminth-virus co-infections we first infected C57BL/6 mice with *L*. *sigmodontis* by exposing the animals to infected mites. 14 days later, when L4 were present in the pleural cavity, the mice were superinfected with a high dose of FV. The time point of *L*. *sigmodontis* infection was chosen since the IgG response to a model antigen and the proliferation of ovalbumin-specific CD4^+^ TCR transgenic T cells is diminished in day 14 *L*. *sigmodontis-*infected mice [36, 37]. Spleen weight and viral loads in spleen, LN and BM were monitored at day 20 post FV infection (day 34 post *L*. *sigmodontis* infection) (Fig 1A). Spleen weight (Fig 1B) was increased at day 20 p.i. in FV-infected mice and even more pronouncedly in co-infected mice. *L*. *sigmodontis* infection alone did not alter the spleen weight nor the number of spleen cells compared to naïve mice (Fig 1B and 1C). Increased splenomegaly was reflected by increased numbers of splenocytes at day 20 p.i. in co-infected mice compared to FV-infected mice (Fig 1C), while numbers of LN and BM cells were similar between the groups (S2 Fig). Viral loads in spleen, LN and BM were higher in *L*. *sigmodontis*/FV-co-infected mice than in FV-infected mice at day 20 p.i. (Fig 1D). Increased FV loads were still observed in co-infected mice at day 35 p.i, although viral loads had declined in co-infected and FV-infected mice at this later time point versus viral loads observed at day 20 p.i. (S2 Fig). In summary, concurrent *L*. *sigmodontis* infection compromised the control of FV infection. Our findings are consistent with two murine studies analysing the outcome of intestinal nematode infections on the course of viral infections. A pre-existing *Trichinella spiralis* infection suppressed control of murine norovirus replication [41] and reactivation of latent γ-herpesvirus was shown in mice co-infected with *Heligmosomoides polygyrus* [42]. Furthermore, a recently published human study showed an increased risk of acquiring HIV seroconversion in individuals suffering from lymphatic filariasis [12].

**Fig 2:**
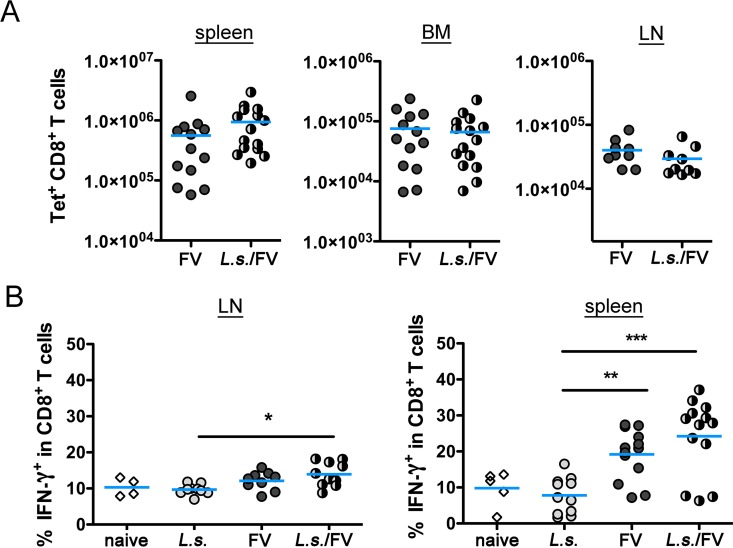
Number of virus-specific CD8^+^ T cells and cytokine response is alike in FV-infected and co-infected mice. C57BL/6 mice were infected for 14 days with *L*. *sigmodontis* and superinfected with FV for an additional 20 days. Control groups were either left uninfected or infected with *L*. *sigmodontis* or FV only. Spleen, BM and LN cells were isolated and analysed by flow cytometry. A) Number of CD8^+^ T cells specific for FV GagL in spleen, BM and LN in FV-infected and co-infected mice at day 20 p.i. LN cells and splenocytes from naïve, *L*. *sigmodontis*-infected, FV-infected and co-infected mice were restimulated *in vitro* with anti-CD3/anti-CD28 for 5 h. Expression of IFN-γ (B) was measured in CD8^+^ T cells. Data are combined from 2–3 experiments (n = 2–5 mice per group and experiment). The line shows the mean and statistical significances are indicated between the groups (*p ≤ 0.05, **p ≤ 0.01, *** p ≤ 0.001).

To elucidate the underlying mechanism we compared the FV-specific immune responses in only FV-infected versus *L*. *sigmodontis*/FV-co-infected mice. Since CD8^+^ T lymphocytes are essential for the control of FV replication during acute infection [43] we first quantified FV-specific CD8^+^ T cells by staining with a class I Tet specific for the dominant FV epitope DbGagL. As expected, Tet^+^ CD8^+^ T cells were absent in lymphoid organs from naïve and *L*. *sigmodontis*-infected mice (S3 Fig), but increased to a similar level in spleen, BM and LN of FV-infected and *L*. *sigmodontis*/FV-co-infected mice (Fig 2A). To analyse the quality of the CD8^+^ T cell response we measured IFN-γ expression in CD8^+^ T cells by intracellular cytokine staining. CD8^+^ splenocytes and LN cells from naïve and *L*. *sigmodontis*-infected mice expressed only low levels of IFN-γ while FV-infected and *L*. *sigmodontis*/FV-co-infected mice had increased frequencies of IFN-γ^+^ CD8^+^ T cells (Fig 2B). Thereby the increase of IFN-γ^+^ expression was more pronounced in CD8^+^ T cells from the spleen than in the LN. Overall, the IFN-γ^+^ response from *L*. *sigmodontis*/FV-co-infected mice resembled that of only FV-infected mice, suggesting that co-infection did not impair the quantity and quality of the CD8^+^ T cell response to FV infection.

Osborne et al. demonstrated reduced numbers and impaired function of virus-specific CD8^+^ T cells in *Trichinella spiralis*-norovirus co-infected mice that correlated with increased viral loads [41]. In our study we observed similar numbers of Tet^+^ CD8^+^ T cells and no differences in the IFN-γ^+^ response of CD8^+^ T cells in general. We analysed the cytokine response of all CD8^+^ T cells since Tet^-^ T cells contribute to the protective immune response against FV as well. Due to this technical difference and a lack of FV-specific peptides for restimulation we cannot exclude that some aspects of the FV-specific CD8^+^ T cell response were impaired in co-infected mice. In a previous study we observed an impaired CD8^+^ T cell response to a vaccine against the liver stage of *Plasmodium berghei* in *L*. *sigmodontis*-infected BALB/c mice [35]. Implementation of a more potent vaccine regime using live Salmonella thereby restored the induction of *plasmodium*-specific CD8^+^ T cells in *L*. *sigmodontis*-infected mice [35] indicating that *L*. *sigmodontis* infection might dampen CD8^+^ T cell responses under certain circumstances. However, strong stimuli such as prime boost immunizations or virus infections might overcome the helminth-induced suppression of CD8^+^ T cell responses.

**Fig 3:**
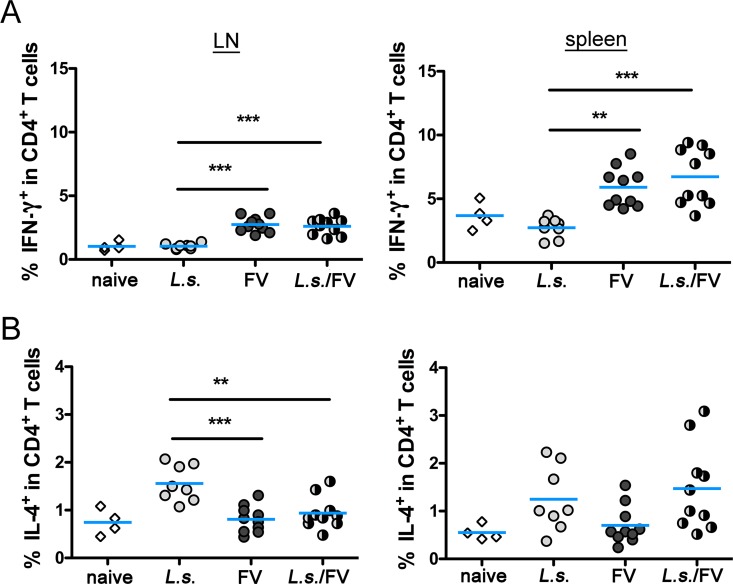
CD4^+^ T cell cytokine response from co-infected mice resembles cytokine expression from FV-infected mice. C57BL/6 mice were infected for 14 days with *L*. *sigmodontis* and superinfected with FV for an additional 20 days. Control groups were either left uninfected or infected with *L*. *sigmodontis* or with FV only. Spleen and LN cells were isolated, restimulated for 5 h with anti-CD3/anti-CD28 and analysed by flow cytometry. Expression of IFN-γ (A), and IL-4 (B) was measured in CD4^+^ T cells. Data are combined from 2 experiments (n = 2–5 mice per group and experiment). Each data point represents an individual mouse. The line shows the mean and statistical significances are indicated between the groups (*p ≤ 0.05, **p ≤ 0.01, *** p ≤ 0.001).

We have previously shown that infection with *L*. *sigmodontis* suppressed the expansion of CD4^+^ T cells recognizing helminth-unrelated antigens, such as ovalbumin or keyhole limpet hemocyanin [34, 36, 37]. During FV infection protective CD4^+^ T cells might either act as T helper cells or exhibit anti-viral cytotoxicity against FV infected cells [26]. Therefore we compared the cytokine response, activation and phenotype of CD4^+^ T cells in spleen and LN during helminth/FV-co-infection. Expression of IFN-γ (Fig 3A) in CD4^+^ T cells reflected the cytokine response observed for CD8^+^ T cells (Fig 2B). CD4^+^ T cells from FV-infected and co-infected mice expressed higher levels of IFN-γ than naïve or *L*. *sigmodontis*-infected mice. Again the expression of cytokines was more pronounced in spleen cells than in LN cells as observed for CD8^+^ T cells. The expression of IL-4, a cytokine that is expressed by follicular T helper cells and Th2 cells, revealed differences between LN and splenic CD4^+^ T cells (Fig 3C). In the lymph nodes, CD4^+^ T cells from *L*. *sigmodontis*-infected mice showed the highest expression of IL-4, while frequencies were significantly lower in CD4^+^ T cells from naïve, FV-infected and co-infected mice. We recorded no statistically significant differences in the expression of IL-4 in CD4^+^ T cells derived from the spleen. Collectively, the analysis of the CD4^+^ and CD8^+^ T cell cytokine responses in LN and spleen revealed a similar IFN-γ expression in FV-infected and co-infected mice. In contrast, the IL-4 expression was suppressed in FV-infected and co-infected mice compared to *L*. *sigmodontis*-infected mice, selectively in the LN but not in the spleen. However, since IL-4 does not alter the course of FV infection [23], a contribution of this cytokine to impaired virus control in co-infected mice is unlikely. In summary the obtained cytokine data rather suggest that FV-induced Th1 polarization outcompeted the pre-existing helminth-induced Th2 polarization as observed for *Schistosoma mansoni*/lymphocytic choriomeningitis virus co-infection [44].

**Fig 4:**
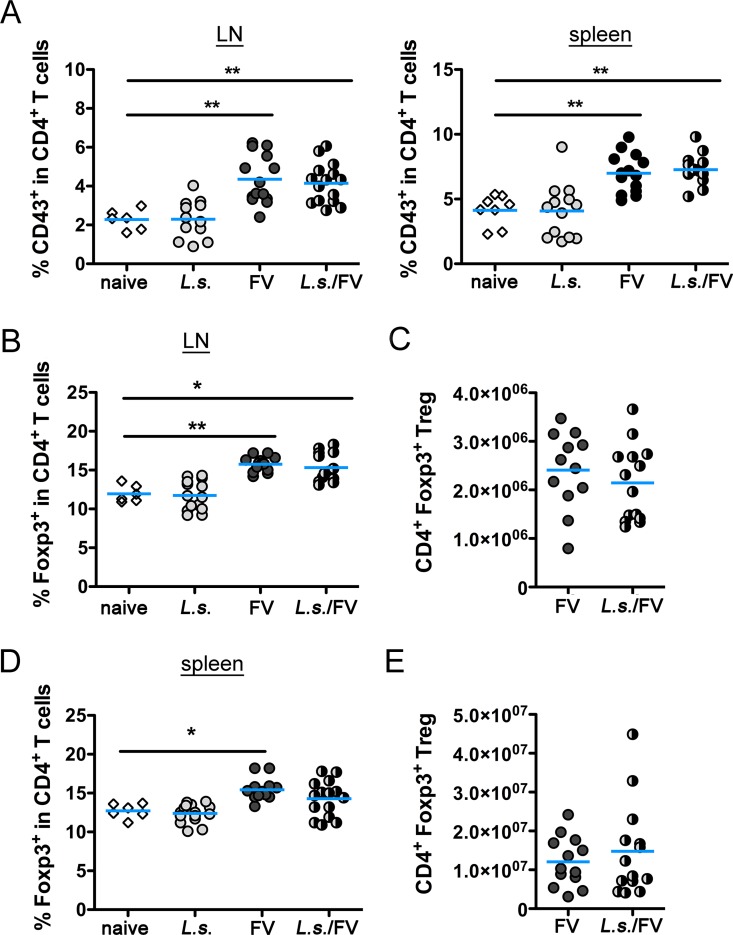
Activation of CD4^+^ T cells and induction of Treg is similar in FV-infected and co-infected mice. C57BL/6 mice were left naïve or infected with FV or *L*. *sigmodontis* or co-infected with *L*. *sigmodontis* and FV. LN and spleen cells were isolated at day 20 p.i. and analysed by flow cytometry. A) Expression of CD43 in CD4^+^ T cells in LN and spleen. Frequencies of Treg in LN (B) and spleen (D) from naïve, *L*. *sigmodontis*-infected, FV-infected and co-infected mice. Absolute numbers of Treg in FV-infected and co-infected mice in LN (C) and spleen (E). Data are combined from 2–4 experiments (n = 2–5 mice per group and experiment). Each data point represents an individual mouse. The line shows the mean and statistical significances are indicated between the groups (*p ≤ 0.05, **p ≤ 0.01).

Next, we measured the activation of CD4^+^ T cells by flow cytometry. *L*. *sigmodontis* infection alone did not induce an activation of CD4^+^ T cells compared to naïve mice, while CD4^+^ T cells from only FV-infected and *L*. *sigmodontis*/FV-co-infected mice displayed a significant upregulation of CD43. The magnitude of the CD4^+^ T cell activation was similar between the two FV-infected groups (Fig 4A). The analysis of CD44 expression and downregulation of CD62L as additional activation markers, as well as the upregulation of Ki-67 as an indicator for T cell proliferation revealed similar results (S4 Fig).

The induction of Foxp3^+^ Tregs favours pathogen survival in both viral and helminth infections [45–47]. In *L*. *sigmodontis* and FV infections, the expansion of Tregs is restricted to the site of infection [46, 48, 49]. To analyse whether *L*. *sigmodontis* and FV synergistically alter Treg responses, we measured percentages and numbers of Foxp3 in CD4^+^ T cells in infected lymph nodes and spleens. Both frequencies and absolute numbers of Foxp3^+^ CD4^+^ Treg were similar in naïve and only *L*. *sigmodontis*-infected mice in spleen (Fig 4B and 4C) and LN (Fig 4D and 4E). Infection with FV induced an expansion of Foxp3^+^ CD4^+^ T cells in both organs, irrespective of pre-existing *L*. *sigmodontis* infection (Fig 4B–4E). We have previously shown that Treg numbers increase during FV infection and interfere with the immune control of the virus during the late phase of the acute infection [39, 46]. Since the expansion of Treg was not exaggerated in *L*. *sigmodontis*/FV-co-infected mice compared to FV infection alone, Foxp3^+^ Treg most likely did not contribute to an impaired control of FV in co-infected mice. In line with this finding, the proliferation of ovalbumin-specific T cells and Ig responses to model antigens were not restored after depletion of Treg in *L*. *sigmodontis*-infected mice [34, 37].

**Fig 5:**
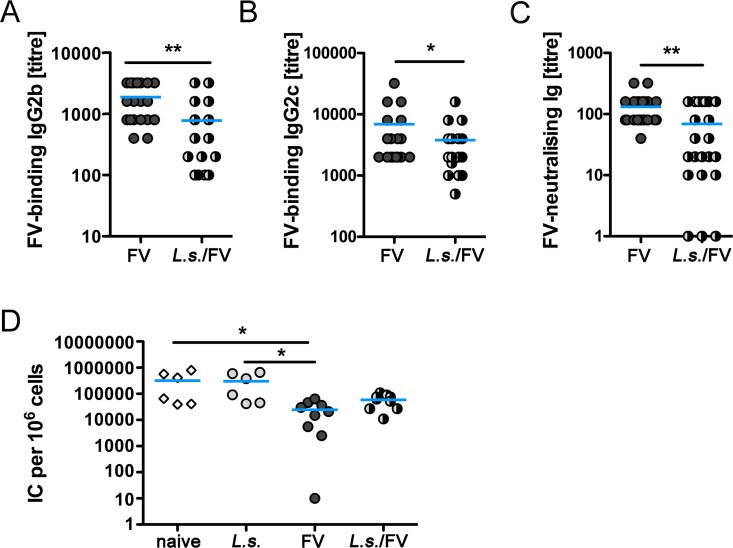
Impaired anti-FV humoral response in co-infected mice. FV-specific IgG2b (A) and IgG2c (B) and FV-neutralising Ig (C) was measured in the serum of day 20 FV-infected or *L*. *sigmodontis*/FV-co-infected mice. D) Recipient mice were infected with FV for 3 days followed by injection of immune (from FV-infected and co-infected mice) and control sera (from naïve and *L*. *sigmodontis*-infected mice). Viral loads in the BM were determined at day 7 p.i. Shown are combined results from 2–5 experiments (n = 3–5 mice per group and experiment). Each data point represents an individual mouse. The line shows the mean and statistical significances are indicated between the groups (*p ≤ 0.05, **p ≤ 0.01).

Recovery from acute FV infection is based on the concerted action of CD4^+^ T cells, CD8^+^ T cells and antibodies [21]. Since CD8^+^ and CD4^+^ T cell responses were similar in FV-infected and co-infected mice, we next analysed the humoral response against the FV helper virus F-MuLV. Protective humoral responses during FV infection are mainly mediated by antibodies of the IgG2 subclass [50] and a beneficial effect of neutralising antibodies in the control of FV was described before [27–29, 51–53]. Thus, we measured F-MuLV-specific IgG in the serum of FV-infected and *L*. *sigmodontis*/FV-co-infected mice by ELISA. FV-specific IgG1 was not detectable at day 20 p.i. Interestingly, co-infected mice had significantly reduced titres of FV-specific IgG2b and IgG2c compared to only FV-infected mice at day 20 p.i. (Fig 5A and 5B). To assess whether this quantitative difference correlated with qualitative differences in the antibody responses, we compared the FV neutralisation capacity of immune sera from day 20 FV-infected and co-infected mice using a complement-dependent neutralisation assay. Sera from co-infected mice had a significantly reduced virus-neutralising capacity compared to FV-infected mice (Fig 5C). To test the clinical relevance of the reduced IgG2 titre and the *in vitro* neutralisation [28, 29] we performed serum transfer experiments. Mice, that received sera from FV-infected animals displayed statistically significant lower viral loads in the BM than mice receiving control sera from naïve or *L*. *sigmodontis*-infected (Fig 5D) animals. Transfer of immune sera from *L*. *sigmodontis*/FV-co-infected mice, by contrast, did not statistically significant reduce the viral load, reinforcing the notion that impaired control of FV in co-infected mice was due to reduced antibody titres. However, viral loads were diminished by trend in mice receiving sera from co-infected mice compared to control sera indicating that the reduced amount of FV-specific antibodies still present in the immune serum of *L*. *sigmodontis*/FV-co-infected mice is nonetheless beneficial for the host. We did not address the mechanism leading to diminished anti-FV antibody responses in the current study. However, the observation that *L*. *sigmodontis*/FV-co-infected mice display decreased FV-specific antibody responses confirm our previous studies showing that infection with *L*. *sigmodontis* drastically reduced IgG responses to a model antigen immunization in BALB/c [34] and C57BL/6 mice [36, 37]. Regarding the mechanism, we showed that reduced IgG responses to model antigen immunization were associated with reduced numbers and frequencies of model antigen-induced follicular T helper cells (Tfh) [34], that are required for a class switch and production of neutralising high affinity antibodies. It was not possible to distinguish FV-specific and *L*. *sigmodontis*-specific Tfh using common markers such as CD44, PD-1 and CXCR5, in the current study, since Tfh are long-lived [54] and infections with *L*. *sigmodontis* and FV both induce germinal centre reactions in the spleen [34, 52].

**Fig 6:**
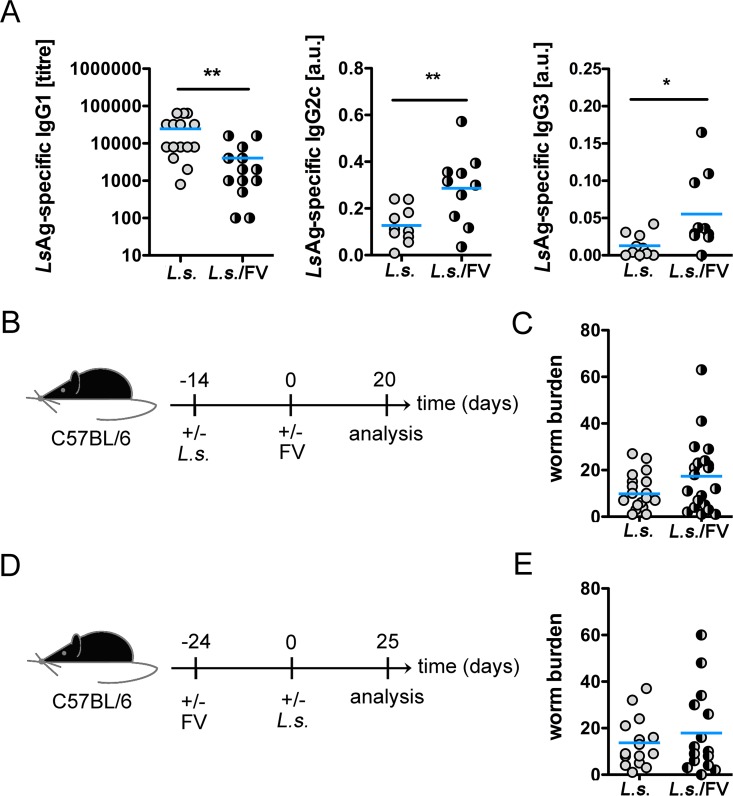
FV infection impacts the *L*. *sigmodontis*-specific humoral immune response, but not the clearance of *L*. *sigmodontis*. C57BL/6 mice were infected for 14 days with *L*. *sigmodontis* and superinfected with FV for additional 20 days. Control groups were either left uninfected or infected with *L*. *sigmodontis* or FV only. A) *Ls*Ag-specific IgG1, IgG2c, and IgG3 was analysed in the sera from *L*. *sigmodontis*-infected and co-infected mice. B) Experimental setup. C) Worm burden at day 34 post *L*. *sigmodontis* infection. D) Experimental setup: Mice were infected for 24 days with FV, followed by infection with *L*. *sigmodontis* for further 25 days. E) Worm burden at day 25 post *L*. *sigmodontis* infection in *L*.*sigmodontis*-infected and co-infected mice. Shown are combined results from 2–4 experiments (n = 4–5 mice per group and experiment). Each data point represents an individual mouse. The line shows the mean and statistical significances are between the indicated groups (*p ≤ 0.05, **p ≤ 0.01).

Since our cytokine data suggest a FV-induced skewing of the immune response towards a proinflammatory cytokine response we analysed whether the FV-infection has an impact on the humoral immune response and eradication of *L*. *sigmodontis*. We found diminished Th2-associated *Ls*Ag-specific IgG1 titres in co-infected mice, while Th1-associated *Ls*Ag-specific IgG2c and IgG3 titres were clearly increased in co-infected mice in comparison to *L*. *sigmodontis*-infected mice (Fig 6A). Thus, FV infection polarizes the humoral immune response against *L*. *sigmodontis* to a more proinflammatory response. Despite the differences in the *L*. *sigmodontis*-specific humoral immune responses we recorded equal numbers of young adults at day 34 post *L*. *sigmodontis* infection, i.e. day 20 post FV infection, in *L*. *sigmodontis*-infected and co-infected mice (Fig 6B and 6C). However, since a higher prevalence of intestinal nematodes has been described in HIV^+^ pregnant women [55], we further analysed a possible impact of a pre-existing FV-infection on the worm burden. To this end, mice were first infected for 24 days with FV followed by infection with *L*. *sigmodontis* (Fig 6D). Despite the pre-existing FV infection, the worm burden was alike in co-infected mice compared to *L*. *sigmodontis*-infected mice (Fig 6E).

In summary, infection with FV, albeit inducing a polarization of the *Ls*Ag-specific humoral immune response, did not have an impact on the worm burden. By contrast, we show that the altered course of a retroviral infection in the presence of a filarial nematode is linked with reduced virus-specific antibody levels.

Due to their asymptomatic nature many helminth species might impair the course of a viral infection without being diagnosed. In this context, our data highlight the importance of deworming programs or the development of vaccines against helminths in developing countries where the incidence of HIV/filarial co-infections is high.

## Supporting Information

S1 Fig Gating strategy. **C57BL/6 mice were either naive, infected with *L*. *sigmodontis* or FV, or co-infected with *L*. *sigmodontis* and FV.** At day 20 days post FV infection lymph nodes were removed and stained with Fixable Viability Dye (Affymetrix eBioscience) according to the manufacturers’ instructions to exclude dead cells. For surface staining, cells were stained with anti-mouse CD4-AF680 (clone: RM4-5), anti-mouse CD8-AF488 (clone: 53–6.7), anti-mouse CD62L BV510 (clone: MEL14). For detection of FV-specific CD8^+^ T cells, cells were stained with PE-labelled MHC class I H2-D^b^ tetramers specific for FV GagL peptide. Representative dot blots showing the gating strategy (A) for expression of Tet^+^ cells in the CD8^+^ T cell gate (B) and expression of activation markers such as CD62L in the CD4^+^ T cell gate (C).
(TIF)

S2 Fig Number of BM and LN cells and viral loads. **C57BL/6 mice were infected for 14 days with *L*. *sigmodontis* (*L*.*s*.) and superinfected with FV for additional 20 days (A+B) or 35 days (C).** Control groups were infected with FV only. Numbers of BM cells (A) and numbers of LN cells (B) at day 20 p.i. Viral loads were determined in spleen, BM, and LN cells (C). Each data point represents an individual mouse. Data are combined from 2–3 experiments (n = 4–5 mice per group and experiment). The line shows the mean and statistical significances are indicated between the groups (*p ≤ 0.05).
(TIF)

S3 Fig Frequencies of virus-specific CD8^+^ T cells. C57BL/6 mice were infected for 14 days with *L*. *sigmodontis* (*L*.*s*.) and superinfected with FV for additional 20 days. Control groups were either left uninfected or infected with *L*. *sigmodontis* or FV only. Frequencies of CD8^+^ T cells specific for FV GagL in spleen (A), BM (B) and LN (C). Data are combined from 3 experiments (n = 2–5 mice per group and experiment). The lines show the mean.
(TIF)

S4 Fig Expression of CD44, CD62L and Ki-67 by CD4^+^ T cells. At day 20 days post FV infection LN and spleens from naive, *L*. *sigmodontis*-, FV- and *L*. *sigmodontis*/FV-co-infected mice were removed and stained with Fixable Viability Dye (Affymetrix eBioscience) according to the manufacturers’ instructions to exclude dead cells. For surface staining, cells were labelled with anti-mouse CD4 AF680 (clone: RM4-5), anti-mouse CD62L BV510 (clone: MEL14) and anti-mouse Ki-67 PE-Cy7 (clone: SolA15). Statistical analysis of CD44 (A), CD62^low^ and Ki-67 expression by CD4^+^ T cells in LN and spleen. Data are combined from 3 experiments (n = 2–5 mice per group and experiment). Each symbol represents a single mouse. *p ≤ 0.05, **p ≤ 0.01.
(TIF)
