# Supplementary figures and images for: Filariae-Retrovirus Co-infection in Mice is Associated with Suppressed Virus-Specific IgG Immune Response and Higher Viral Loads
Source: PLoS Negl Trop Dis. 2016 Dec 6;10(12):e0005170. doi: 10.1371/journal.pntd.0005170 (PMC5140070; doi:10.1371/journal.pntd.0005170)

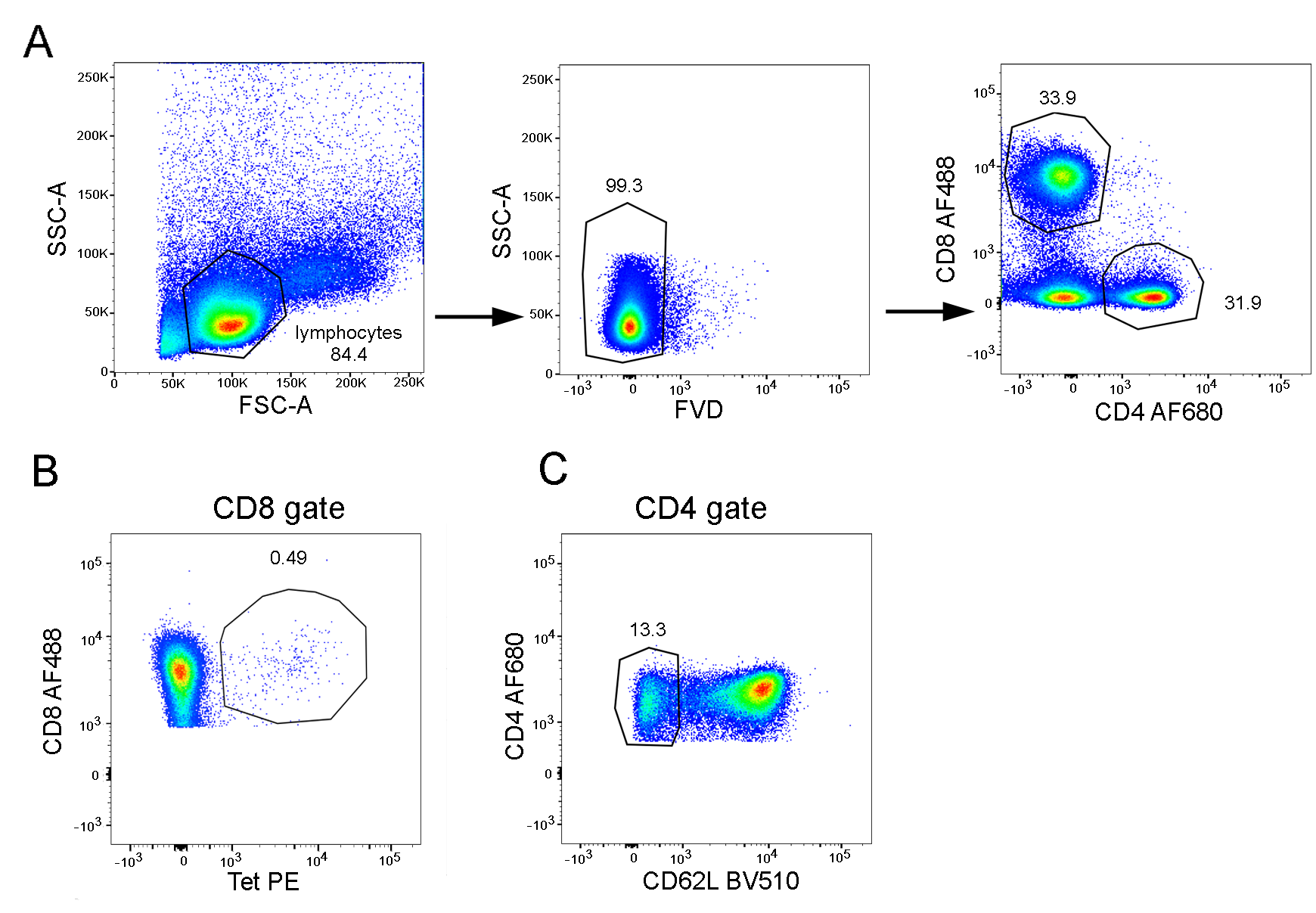

Supplement: S1 Fig — C57BL/6 mice were either naive, infected with L. sigmodontis or FV, or co-infected with L. sigmodontis and FV. At day 20 days post FV infection lymph nodes were removed and stained with Fixable Viability Dye (Affymetrix eBioscience) according to the manufacturers’ instructions to exclude dead cells. For surface staining, cells were stained with anti-mouse CD4-AF680 (clone: RM4-5), anti-mouse CD8-AF488 (clone: 53–6.7), anti-mouse CD62L BV510 (clone: MEL14). For detection of FV-specific CD8+ T cells, cells were stained with PE-labelled MHC class I H2-Db tetramers specific for FV GagL peptide. Representative dot blots showing the gating strategy (A) for expression of Tet+ cells in the CD8+ T cell gate (B) and expression of activation markers such as CD62L in the CD4+ T cell gate (C). (TIF) [file pntd.0005170.s001.tif]

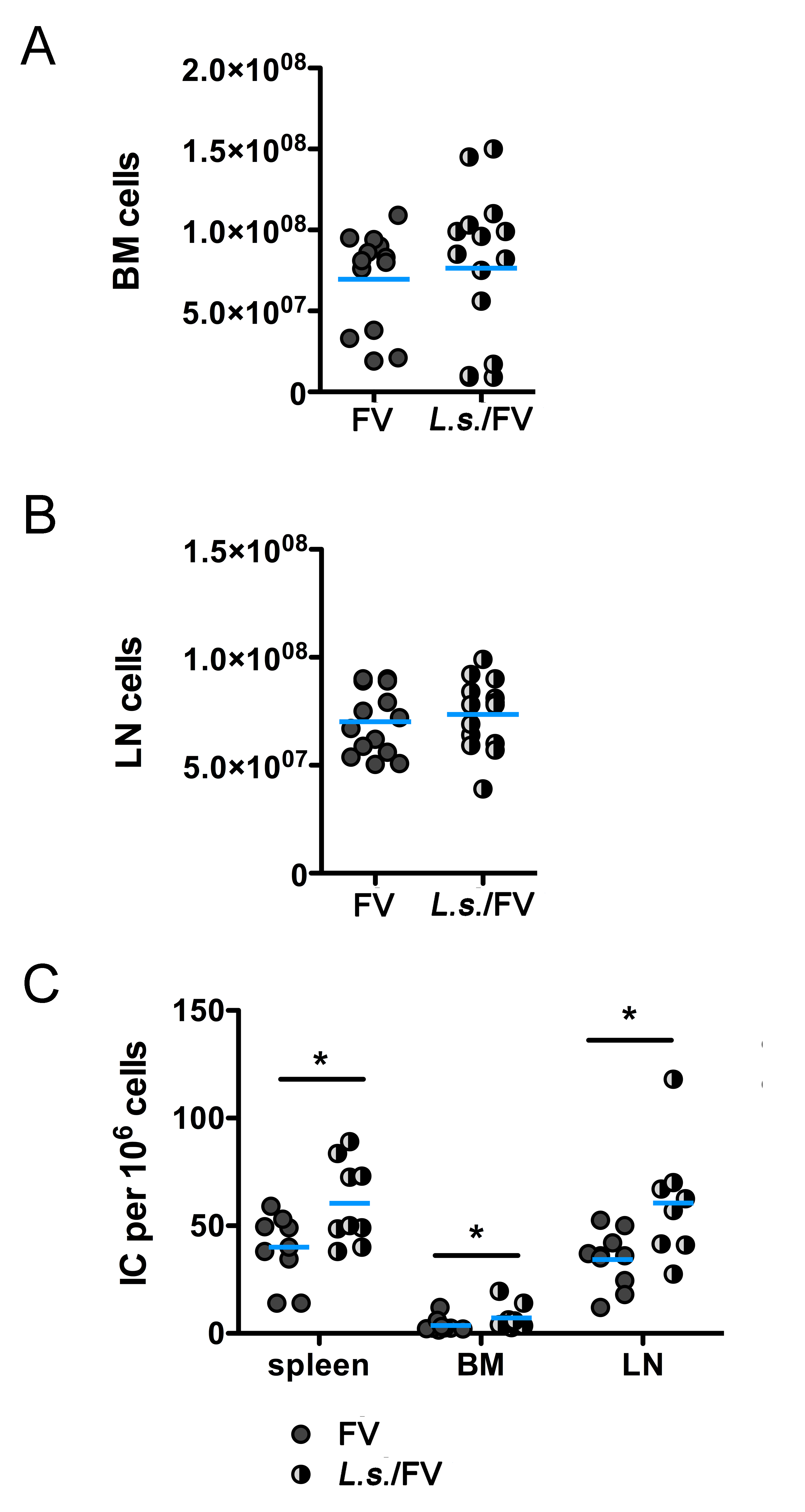

Supplement: S2 Fig — C57BL/6 mice were infected for 14 days with L. sigmodontis (L.s.) and superinfected with FV for additional 20 days (A+B) or 35 days (C). Control groups were infected with FV only. Numbers of BM cells (A) and numbers of LN cells (B) at day 20 p.i. Viral loads were determined in spleen, BM, and LN cells (C). Each data point represents an individual mouse. Data are combined from 2–3 experiments (n = 4–5 mice per group and experiment). The line shows the mean and statistical significances are indicated between the groups (*p ≤ 0.05). (TIF) [file pntd.0005170.s002.tif]

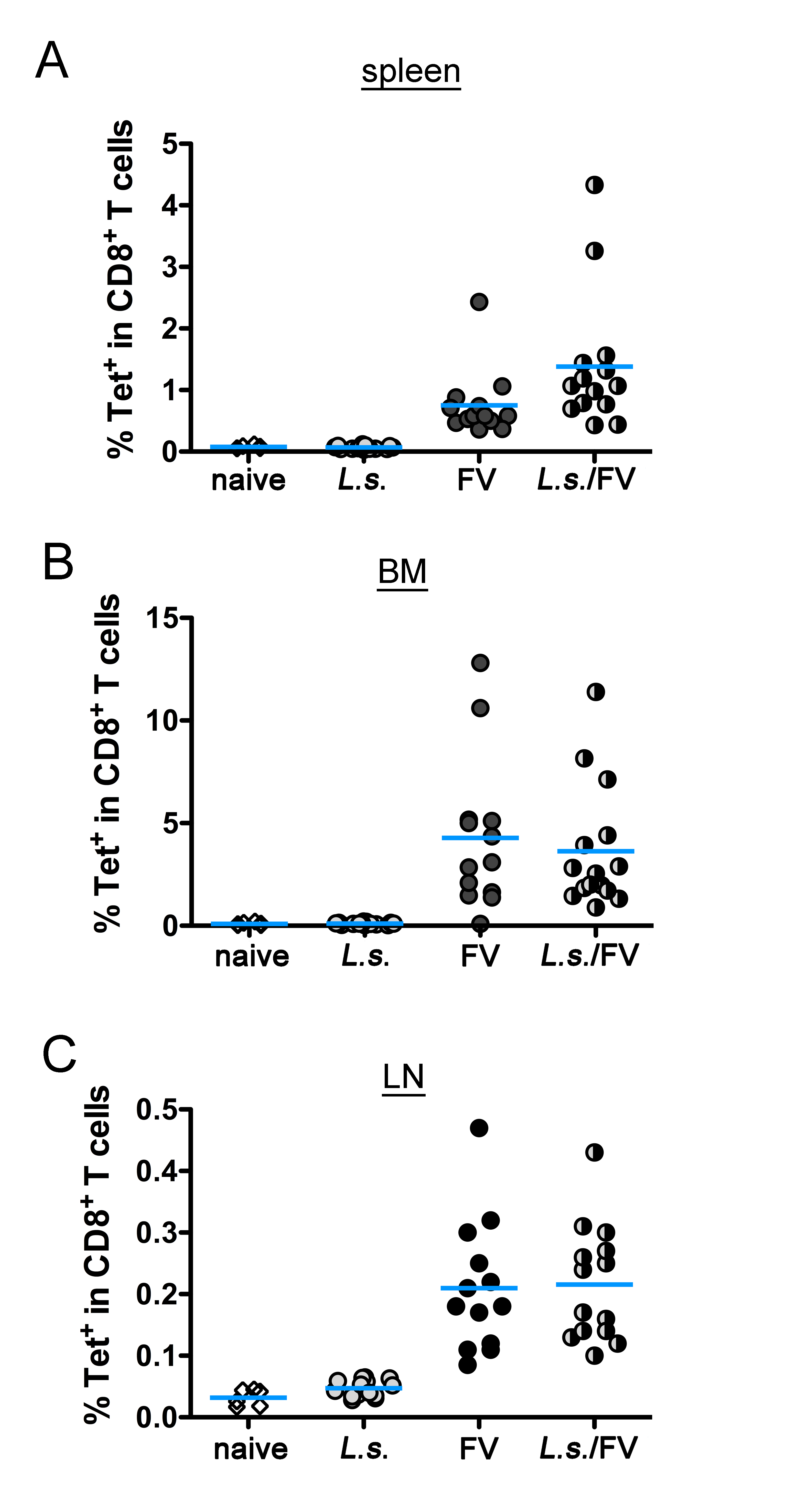

Supplement: S3 Fig — C57BL/6 mice were infected for 14 days with L. sigmodontis (L.s.) and superinfected with FV for additional 20 days. Control groups were either left uninfected or infected with L. sigmodontis or FV only. Frequencies of CD8+ T cells specific for FV GagL in spleen (A), BM (B) and LN (C). Data are combined from 3 experiments (n = 2–5 mice per group and experiment). The lines show the mean. (TIF) [file pntd.0005170.s003.tif]

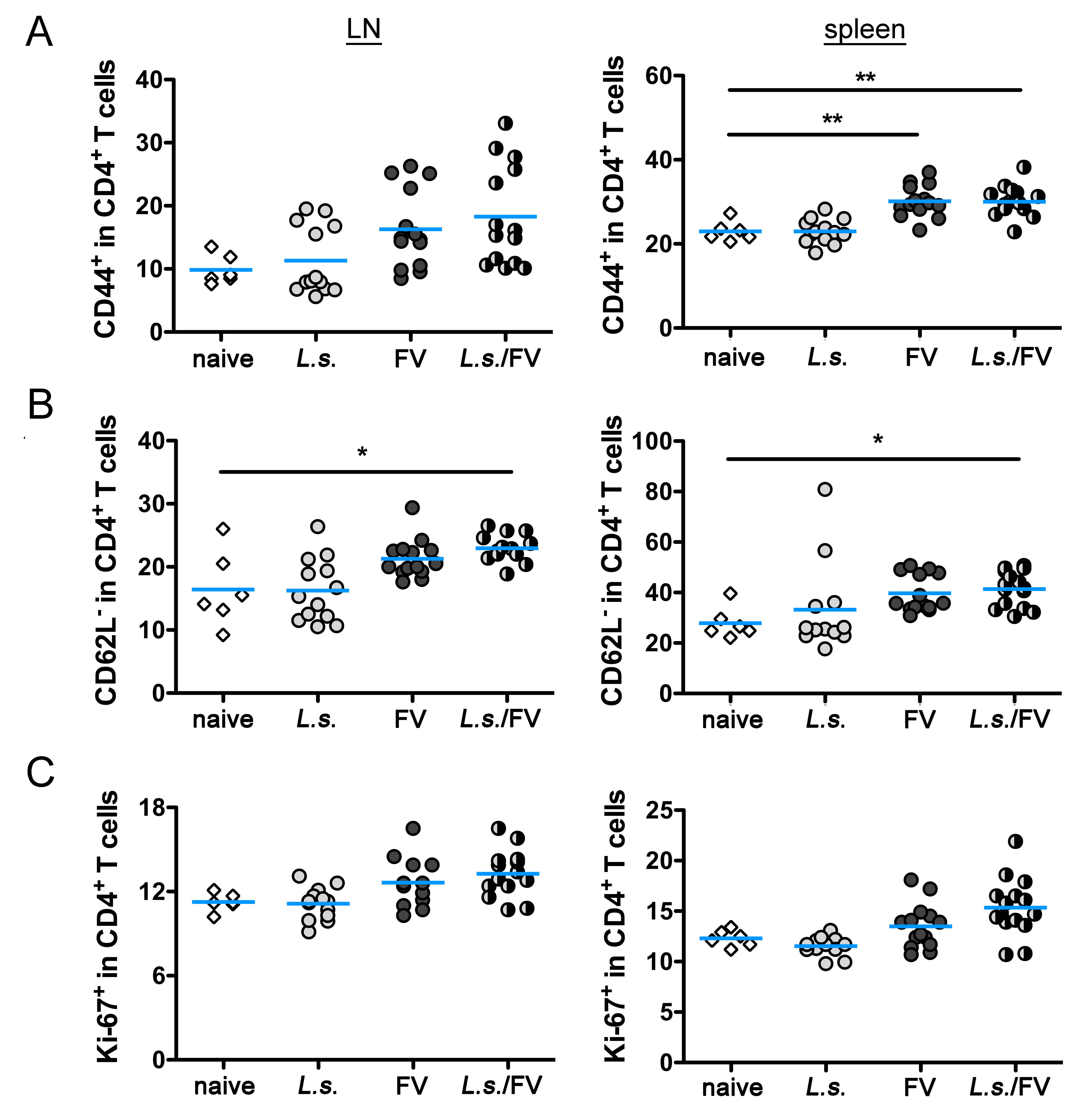

Supplement: S4 Fig — At day 20 days post FV infection LN and spleens from naive, L. sigmodontis-, FV- and L. sigmodontis/FV-co-infected mice were removed and stained with Fixable Viability Dye (Affymetrix eBioscience) according to the manufacturers’ instructions to exclude dead cells. For surface staining, cells were labelled with anti-mouse CD4 AF680 (clone: RM4-5), anti-mouse CD62L BV510 (clone: MEL14) and anti-mouse Ki-67 PE-Cy7 (clone: SolA15). Statistical analysis of CD44 (A), CD62low and Ki-67 expression by CD4+ T cells in LN and spleen. Data are combined from 3 experiments (n = 2–5 mice per group and experiment). Each symbol represents a single mouse. *p ≤ 0.05, **p ≤ 0.01. (TIF) [file pntd.0005170.s004.tif]
